# Surviving a crisis: A multilevel model of leadership styles, employees’ psychological capital and organizational resilience

**DOI:** 10.1371/journal.pone.0318515

**Published:** 2025-02-06

**Authors:** Faith Njaramba, John Olukuru

**Affiliations:** Strathmore Business School, Strathmore University, Nairobi, Kenya; Guangxi Normal University, CHINA

## Abstract

Building on the emerging research on organizational resilience in crisis, we tested a model of transformational and directive leadership styles and their association with organizational resilience in a crisis via employees’ psychological capital. Three hundred and one small and medium sized enterprises in Kenya provided usable survey data. The hypotheses were tested using multilevel structural equation modelling (MSEM) technique. The results show that both transformational and directive leadership styles were significant and positive predictors of organizational resilience through the mediating role of employees’ psychological capital. From a practical perspective, as SMEs struggle to be resilient during crises, leaders should adopt effective leadership styles such as transformational and directive and also consider their employees’ psychological experience of a crisis.

## Introduction

Crises refer to occurrences that threaten the fundamental operation or the viability of an organization [1]. Although crises can have significant impact on organizational survival, they cannot be predicted with accuracy, nor can they be avoided [2]. Crises are increasing in frequency and complexity and the latest crises, such as the COVID-19 pandemic, confronted all leaders [3,4]. And as Riggio and Newstead [2] argue, there is no situation where leadership is most important. Since many crises tend to occur quickly and then are resolved, leadership research has paid relatively little attention to crisis-leadership.

The COVID-19 pandemic provided a unique opportunity to study leadership in a crisis [3,4]. This pandemic was an acute organizational stressor that impacted personal, social and organizational resources in destabilizing ways [5]. Although previous studies have explored the link between leadership style and organizational performance [6,7], few of these have studied these issues during crises, hence, theory regarding leadership effectiveness is nascent. Additionally, a dearth of studies has investigated the theoretical links regarding how leadership relates to employee resources, which can be critical for organizational effectiveness and at the very least, survival [8]. Of the studies that exist, few have studied small and medium sized enterprises (SMEs) [9]. SMEs are important because they not only form the highest number of organizations worldwide, but they can be especially vulnerable to crises because of a lack of slack resources [10]. Last, while most studies have taken place in more developed economies, hardly any research has been conducted in developing countries such as those in Africa which is the continent where the pandemic creeped in last but had lasting effects on many businesses [3,11].

The purpose of the present study is therefore to investigate the link between leadership style and organizational ability to cope during a crisis. We focus on the COVID-19 crisis because it has been the largest crisis worldwide that businesses can learn from [12]. Specifically, we explore how two leadership styles namely transformational and directive leadership affect the organization’s ability to be resilient during a crisis. Transformational leadership is the most widely studied leadership style during crises and it resonates highly with employees [13,14]. Directive leadership on the other hand is important in practice for crisis management to provide clear directions, clarity and structure [15,16]. We draw and build upon the job demands resources (JD-R) theory to explore how the leadership styles relate to the psychological capital of employees to contribute to organizational resilience. In specific, JD-R theory is applied to bring out the two leadership styles as work resources which together with employees’ psychological capital, which is a personal resource [17], enable organizations to cope with the demands of a crisis.

Our research offers four important contributions. First, we identify and explore a mechanism that can help explain how leadership styles can contribute to organizational resilience, namely, through employees’ psychological capital. The present study answers a call to highlight the interdependent nature of organizational resilience by elaborating on the importance of the interplay of leadership-related and employee-related factors in building organizational resilience [18]. This interdependence can offer practical insights into how resilience can be established in organizations. The mediating mechanism also points to the importance of considering the psychological experience of employees during a crisis. Second, the present study integrates micro- and macro-level organizational behavior variables to address a dearth of multilevel research on the antecedents of organizational resilience [19]. Micro level variables serve to address the behavior and contribution of individuals in a business, while the macro-level variables address the organization as a whole [20]. As such, we answer a call to take on a multilevel perspective to understand the resources that leaders may activate to achieve resilience during a major external crisis [21]. This brings out the importance of expressing resilience at several levels and identifying the drivers (leaders) needed to cultivate such resilience [22]. The present paper tests and contributes to the JD-R theory that enables us to not only model organizational life at various levels but also integrate personal resources into organizational resources [17]. Third, the present study also contributes to practice, in particular to SMEs by providing empirical evidence of which leadership style is most relevant for organizational resilience in a crisis [18]. Leadership is a frugal resource that is within the reach of most SMEs and therefore it is important to understand which styles can make a difference in resilience during crises [23]. Fourth, the present study makes a contribution by drawing on data from SMEs in Kenya, a developing country. There is a dearth of research from developing countries’ yet we know that resilience is not a ‘one size fits all’ issue [24,25].

Given this background, our research aimed to unearth the questions below:

RQ1. What is the relationship between leadership styles (transformational and directive) and organizational resilience?

RQ2. How does leadership style relate to resilience? Here we explore the mediating effects of psychological capital in the relationship between leadership style and organizational resilience.

In the next sections we provide a discussion of theory and hypotheses which we subsequently test using defined research methods. Finally, we conclude with a discussion of the results, implications and indications for the future expansion of this study.

### Theoretical development

The present study applied the lens of the job demands resources theory to analyze the research questions as expounded below.

#### Job-demands resources theory.

According to the job demands resources (JD-R) theory, work environments are usually composed of job demands and resources [26]. Job demands constitute the psychological, physical, social and organizational aspects of a job which require sustained effort [26]. Job demands, usually expressed by factors such as work overload, role conflict and role ambiguity, typically exhaust employee’s mental and physical resources [27]. Job resources on the other hand, are the aspects of a job that help employees deal with job demands [17]. Job resources constitute factors such as performance feedback, social support and skill variety which satisfy employees’ basic psychological needs, including the need for competence, relatedness, and autonomy [17]. A recent extension of the resource dimension of JD-R theory was made thereby including personal resources alongside job resources [26]. Personal resources usually refer to people’s beliefs about how much control they have over their environment and include aspects such as self-efficacy, hope, resilience and optimism [26]. Personal resources spiral and affect organizational outcomes [26].

JD-R theory is predominantly used in literature to analyze how the work environment affects well-being and performance [28]. JD-R has proven its adaptability across countries, sectors and occupational groups though it is less commonly applied in studies from developing countries [29,30]. SMEs in developing countries encountered significant changes in job conditions including having significantly more job demands and coupled with less job resources during the COVID-19 pandemic [11] and hence it is important to apply JD-R theory in this context to understand how the crisis environment affected the resilience of SMEs.

In the present study we apply the JD-R theory to argue that the COVID-19 crisis provided an environment of immense job demands to employees in the form of pressure from increased workload, altered work contexts, uncertainty and increased dismissal [31] and hence they needed effective leaders to boost their personal resources so that they all could contribute to organizational resilience. Indeed, past literature recognizes that organizational resilience is the result of dynamics that create or maintain cognitive, emotional, relational or structural resources within an organization [32].

On the basis of the JD-R theory, we see that leaders can facilitate organizations to be resilient in two ways. By directly influencing job demands and resources or by influencing the impact of job demands and resources on employees’ well-being [28]. When employees experience a high workload, leaders, through their leadership style, can help by reducing this job demand directly through deciding what the priorities are for the organization and which work aspects can safely be ignored [28]. Leaders can also reduce job demands indirectly. For instance, leaders can increase job autonomy by letting employees decide when and where to work in order to perform optimally during demanding times [28].

Lastly, in the present study we use JD-R theory to view resilience as the resource-based capability of a firm to use personal and job resources to survive and thrive in a crisis. We apply the recent extension of the JD-R theory to add that psychological capital, which is a personal resource, builds up the repository of job resources. Individuals having a greater amount of personal resources are less prone to resource depletion when facing adversity in the workplace [33] and leaders act as one of the sources of employees’ resources to maintain/build their psychological capital in order to deal with job demands [34]. Lastly, psychological capital has been found in previous studies to have a positive effect on work outcomes such as work performance [33] hence our need to test its relationship with organizational resilience. Our conceptual framework is provided in S1 Fig.

### Hypotheses development

#### Transformational leadership and organizational resilience.

Drawing from JD-R theory, we argue that transformational leadership is a job resource that reduces job demands during crises hence enabling an organization to be resilient. Organizational resilience is defined as a meta capability that enables firms to cope effectively with unexpected events, bounce back from crises, and even foster future success through the right utilization of resources [27–29].

Transformational leadership, through its four behaviors of individualized consideration, intellectual stimulation, idealized influence and inspirational motivation, serve as important resources that can potentially help an organization to be resilient [35,36]. For instance, through individual consideration leaders are able to support employees by checking on them, responding to them when they ask for help, and complimenting them when they do a good job and this raises the expectations of employees resulting in positive performance outcomes [37]. Intellectual stimulation on the other hand causes followers to question systems, processes and assumptions, thereby causing them to emerge with innovative and creative ideas to solve challenges [38]. Through inspirational motivation of followers, employees can visualize an alluring future during a crisis and set high performance standard while through idealized influence, employees can learn on how to work hard through a painful journey to achieve the idealized picture of the future [35]. Transformational leadership style is indeed engrossed with thoughts of change and is the kind of leadership required to survive volatile, uncertain, complex, and ambiguous business environment [38]. Transformational leadership is particularly effective for SMEs to live to their full potential against all odds by finding innovative ways to beat their challenges [39]. We therefore hypothesize that during a crisis,

H1: Transformational leadership is positively related to organizational resilience.

#### Directive leadership and organizational resilience.

Drawing from the JD-R theory, we view directive leadership as a job resource that reduces job demands during crises by emphasizing clear goal setting, respect for hierarchy, giving feedback and initiating change, all which enable organizational resilience. This type of resilience is in line with Burnard and Bhamra’s [40] conceptualization of organizational resilience as the capability of firms to understand the situation, resist, and respond wisely. Directive leadership emphasizes the initiating nature of a leader [41] which enables them to accurately perceive the situation and respond appropriately leading to higher performance [15,41].

Directive leadership has been found to be effective to ensure performance in unstable contexts and especially for start-ups [42]. Leaders have been reported to being able to boost organizational resilience by directing complex and contradictory tasks [43]. Directive leadership also leads to better outcomes in high-intensity organizational contexts and hierarchically organized communities such as those in many developing countries [44,45]. Directive leadership legitimizes hierarchy which is important for the stability of the organization especially during crises, when there is utmost need for subordinates to respect the position, knowledge and experience of their superiors [15]. Directive leadership could provide much needed clarity and structure during a crisis based on its characteristics but no study to the best of our knowledge has related directive leadership to organizational resilience during a macro crisis. We therefore hypothesize that during a crisis,

H2: Directive leadership is positively related to organizational resilience.

#### Transformational leadership and psychological capital.

Drawing from the JD-R theory, we view transformational leadership as a job resource that enhances psychological capital through increasing an employee’s sense of efficacy, hope, resilience and optimism during crises. During a time of extreme crisis, employees experience uncertainty and transformational leaders could offer confidence and clarity through individualized consideration and intellectual stimulation [37]. Transformational leaders also utilize emotions to convince their employees to engage in positive thinking in terms of developing both a positive vision and new ideas and this increases their optimism [46]. Transformational leadership is also positively associated with subordinates’ self-efficacy whereby employees tend to feel more confident in their abilities to pursue greater goals if they have a transformational leader who leads by counseling, mentoring and coaching [46].

In general when organizations face crises, transformational leadership can make organizational members to believe that the crisis will end soon and hence increase their personal resources through contagion effects from interacting with such a leader [47]. We therefore hypothesize that during a crisis,

H3: Transformational leadership is positively related to employees’ psychological capital.

#### Directive leadership and psychological capital.

Based on the application of the JD-R theory, we view directive leadership as a job resource that reduces job demands during a crisis by setting clear goals, teaching, offering feedback hence enabling employees to feel more hopeful, resilient, efficacious and optimistic. The primary role of a directive leader is to convey to subordinates the methods by which to achieve success [15]. Directive leaders usually assume responsibility in terms of training (or teaching and educating) their subordinates for achievement [15].

Contrary to some literature, directive leadership does not have to be authoritarian whereby emphasis is on absolute control and unquestionable obedience from subordinates [15]. No research to the best of our knowledge has related directive leadership to psychological capital or its elements but based on the view that directive leadership can be beneficial to teach for higher achievement [15], we hypothesize that during a crisis,

H4: Directive leadership is positively related to employees’ psychological capital.

#### Psychological capital and organizational resilience.

Based on the JD-R theory, we hypothesize that psychological capital is part of the resources that are used in organizations to enable employees to contribute to organizational resilience. Similar to job resources, personal resources act as independent predictors of work engagement [48]. Organizational resilience emanates from the organizational sub-units (employees) and represents the ability to cope with adversity by effectively utilizing resources across different levels of the organization [49]. Employees play a critical role in building organizational resilience and hence they need to be empowered to do so [50] by building their psychological capital. Psychological capital is recognized as a positively oriented psychological capacity that is measurable and malleable to enhance workplace performance [51].

Past research has only viewed organizational outcomes such as work commitment and performance as elements that emanate from employees’ psychological capital [49]. But for employees to take on any meaningful endeavor (such as contributing to organizational resilience), they must possess at least some degree of psychological capital to attempt endeavors they do not believe they can do successfully (*self-efficacy*), to walk into what appears as destine (*optimism*), to take the first step into a pathway (*hope*) and to bounce back after dismal failure (*resilience*) [52]. We therefore hypothesize that during a crisis,

H5: Employees’ psychological capital is positively related to organizational resilience.

#### The mediating role of employees’ psychological capital on the relationship between leadership styles and organizational resilience.

Drawing from the JD-R theory, we view psychological capital as a personal resource that can be fostered by leaders thereby mobilizing positive behaviors in an organization that contribute to organizational resilience. Previous research has found that in the face of adversity, psychological capital predicts work engagement since employees are more likely to perceive their control of the situation [53]. During the COVID-19 pandemic, there were many unknowns (unstable economies, mortality) and subordinates really looked to their leaders for inspiration and direction [37]. We propose that positive leadership styles such as transformational and directive styles operate by increasing psychological capital which in turn activates organizational resilience as discussed below.

First, it is known that transformational leaders have the ability to broaden and elevate employees’ confidence to perform beyond the expectations specified in the implicit or explicit exchange agreement [54]. How this happens is through mediating factors. A few mediators between transformational leadership and positive organizational outcomes have been previously studied including affective, motivational, identification, social exchange, and justice enhancement which cover social and psychological resources [55]. Limited research has examined transformational leadership in extreme crises and the conditions under which it maintains its positive effect [37]. We argue that psychological capital is an important mediator in our case to bring in the leader-employee dynamics that shape organizational resilience. In deed Lengnick-Hall et al. [56] asserted that organizational resilience is an outcome that can be developed by capitalizing on the cumulative strengths of both leaders and employees. We therefore hypothesize that during a crisis,

H6: Employees’ psychological capital mediates the positive relationship between transformational leadership and organizational resilience.

Second, we argue that directive leaders who operate by providing followers with the means to achieving goals [15,57] could enhance follower’s psychological capital which enhances organizational resilience. Mechanisms that have been previously studied between directive leadership and organizational outcomes include, commitment which mediated the positive relationship between directive leadership and innovative work behavior [58], promotion and prevention focus which mediated the positive relationship between directive leadership and work role performance [57] and competitive simplification which mediated the positive relationship between directive leadership and financial performance [59]. No study to the best of our knowledge has tested psychological capital as a mediator in the relationship between directive leadership and organizational resilience. It is however important to test psychological capital, as a mediator between directive leadership and organizational resilience to bring out the potential role of a firm’s internal relational resources. Notably, directive leadership is commonly used in hierarchical cultures, in unstable contexts, and in start-up firms [43–45] which forms the context of this present study. We therefore hypothesize that during a crisis,

H7: Employees’ psychological capital mediates the positive relationship between directive leadership and organizational resilience.

## Methods

### Participants

A cross-sectional survey design was used. The data collection period was between 01/02/2022 and 28/02/2022, when Kenya was facing the fifth wave of COVID-19 infections [60]. A wave refers to a period of increased disease incidence [61]. The unit of analysis for the present study is the firm.

The sample size was 398 SMEs calculated using the Yamane formula [62]. The participating SMEs were selected from a population of 121719 licensed SMEs in Kenya [63] using convenience sampling technique. Convenience sampling is widely used in SME research in Kenya where there is no sampling frame of all SMEs employees [64]. Our selection criteria included picking organizations with between 10–99 employees which define SMEs in Kenya [63].

These SMEs were drawn from three counties including Nairobi, Nakuru and Kiambu which were worst hit by the COVID-19 pandemic [65]. Primary data was collected using structured questionnaires that were administered by a trained research team. A pair of employees who had been working in an SME since the onset of the pandemic and with a boss were requested to fill in a questionnaire each. Multiple respondents per firm serve to increase the reliability of the measures [66]. The study obtained nested data with 301 SMEs providing usable data after data cleaning hence a response rate of 75.62%. All measures were assessed by employee self-report.

### Instruments

Transformational leadership was assessed on a five-point Likert scale using the Multifactor Leadership Questionnaire (MLQ) by Bass and Avolio [67] which consists of twenty measurement items. This is the most widely used tool used to measure transformational leadership and includes four dimensions of idealized influence, inspirational motivation, intellectual stimulation and individual consideration [68]. An example of an item from the MLQ is ‘my boss talks optimistically about the future’. The Cronbach alpha value for transformational leadership in this study was 0.92.

Directive leadership was assessed on a five-point Likert scale using the Directive Leadership Tool (DLT) of Litwin and Stringer which contains seven measurement items [69]. The directive leadership tool was the most widely used measure of directive leadership especially in the pandemic crisis period [57]. An example of an item from the DLT is ‘my boss expects me to follow his/her instructions precisely.’ The Cronbach alpha value for directive leadership in this study was 0.89.

Psychological capital was assessed on a five-point Likert scale using the psychological capital scale (PCQ) with twenty four items [70]. This is the most established measure of psychological capital which has four dimensions including optimism, resilience, hope and self-efficacy [71]. An example of an item from the PCQ is ‘if I should find myself in a jam at work, I could think of many ways to get out of it.’ The Cronbach alpha value for psychological capital in this study was 0.91.

Organizational resilience was assessed on a five-point Likert scale using the short version of the Benchmark Resilience Tool (BRT) by Whitman et al. [72]. This tool contains thirteen measurement items and was widely used to measure organizational resilience during COVID-19 pandemic period [73]. An example of an item from the BRT is ‘we are able to shift rapidly from business-as-usual to respond to crises.’ The Cronbach alpha value for organizational resilience in this study was 0.92.

### Common method variance

In the present study, we took several recommended measures to reduce common method variance [74]. First, the respondents anonymously answered the questionnaire and were assured that no direct reference would be made to them. Second, we used only high quality and validated measures of the variables considered. Thirdly, clear sets of instructions were provided to the respondents with an emphasis that they should rate the items frankly and independent of all other statements.

Additionally, ex-post Harman’s univariate test was used to test whether the data involved in this study contained significant common method bias. The variance explanation rate extracted by one factor was 32.53% which is less than the 50% threshold indicating that there was no significant common method bias [75].

### Control variables

In order to account for possible compounding effects of other factors that could affect the hypothesized relationships, the firm’s sector, mode of working of employees and presence of IT back-up facilities were used as covariates in the analyses. Generally, the service sector was disproportionately affected by the pandemic due to the need to reduce human contact during the pandemic [35]. Additionally, firms that had established remote mode of working tended to be more resilient and the presence of IT back-up facilities also enabled firms to access their customer records and other forms of data that were essential for business continuity [76]. These factors could lead to a higher chance of organizational resilience [73,76] and therefore, their inclusion in the model to account for them was important.

### Data analysis

Before the testing of the hypotheses, the psychometric properties of the tools were ascertained. The reliability of the scales was assessed using Cronbach’s alpha and the values ranged from 0.89 to 0.92. Composite reliability (CR) values ranged from 0.91 to 0.93. Therefore all the constructs met the recommended reliability threshold of above 0.7 [77]. The assessment of skewness and kurtosis was also done and all variables were within the recommended normality thresholds [78] though it is best to invoke non-parametric methods to conduct analyses on any skewness above plus or minus 0.5 [79] such as organizational resilience below. The skewness values were as follows; organizational resilience (−0.55), psychological capital (0.13), transformational leadership (−0.11) and directive leadership (−0.23). The kurtosis values were as follows, organizational resilience (−0.60), psychological capital (−0.71), transformational leadership (−0.96) and directive leadership (−0.84). The predictors maintained variance inflation factors that were below the threshold of 3 [80]. This was all after discarding two aspects of psychological resilience (labelled PSY-RES 4&5) which were highly correlated with other psychological resilience items. Best practice recommends discarding such items [81].

We assessed discriminant and convergent validity of the variables in this present study using heterotrait monotrait ratio (HTMT) and average variance extracted (AVE) respectively [82,83]. All HTMT ratios were within the threshold of below 0.85 [82] as follows: Between directive leadership and psychological capital =  0.31, between transformational leadership and psychological capital =  0.27, between transformational leadership and directive leadership = 0.31, between organizational resilience and psychological capital =  0.28, between organizational resilience and directive leadership =  0.36 and between organizational resilience and transformational leadership 0.31. AVE values were above the 0.50 threshold apart from organizational resilience and transformational leadership which however met the 0.4 threshold plus had good composite reliability values of above 0.70 as recommended in research, hence signifying convergence within scales [35,84–86]. AVE values were as follows: directive leadership =  0.60, organizational resilience =  0.50, psychological capital =  0.35 and transformational leadership =  0.39. The constructs’ Cronbach’s reliability, composite reliability, variance inflation factors, average variance extracted and heterotrait monotrait values between the variables are presented in Table 1.

**Table 1:** Reliability and validity tests results.

Constructs	Cronbach’s alpha	CR	VIF	AVE	1	2	3
**Organizational resilience**	0.92	0.93	–	0.50	–		
**Transformational leadership**	0.92	0.93	2.38	0.39	0.31	–	
**Directive leadership**	0.89	0.91	2.00	0.60	0.35	0.31	–
**Psychological capital**	0.91	0.92	2.59	0.35	0.28	0.27	0.31
**Threshold**	>0.70	>0.70	<3	>0.40	<0.85		

CR, composite reliability; VIF, variance inflation factor; AVE, average variance extracted.

### Quality of the measurement model

A multilevel confirmatory factor analysis was performed using R software (Lavaan), and the results showed that the data structure was fit for further data analysis. We used non-parametric SEM methods throughout the analysis of this nested model and interpreted robust model fit statistics [87]. We generated robust comparative fit index (CFI) and the Tucker-Lewis index (TLI) indices all which compare the fit of the specified model to that of the baseline model (null model) both with a fit threshold of above 0.95. We also obtained the robust root mean square error of approximation (RMSEA) which assesses the goodness of fit per degree of freedom with a fit threshold of below 0.05 and the robust standardized root mean square residual (SRMR) which assesses the average discrepancy between observed and predicted correlations with a fit threshold of less than 0.08 [88,89]. In the present study the robust CFI (0.95), TLI (0.94), RMSEA (0.03) and SRMR (0.04), all indicated excellent fit. The chi square to degrees of freedom ratio was 1.54 which is within the conservative threshold of below 2 [90]. (χ² = 2795.89, df = 1817), further supporting the model’s adequacy.

We proceeded to estimate the structural models to test our hypotheses using Stata software (version 18). We employed generalized structural equation modeling (GSEM) to analyze our structural models due to its greater suitability than structural equation modeling (SEM) for mediation models with individual-level predictors and organizational level outcomes, which are expressed as (1➔1➔2) models [91,92]. 1➔1➔2 models are suitably addressed within the multilevel structural equation modeling (MSEM) framework as compared to multilevel linear regression modeling framework [91]. The MSEM framework is powerful and offers significant advantages, including mitigating the effects of measurement errors due to utilizing latent variables [93], allowing for the estimation of both between- and within-level components of indirect effects without necessitating group-mean centering [91], employing a latent-variable approach to yield less biased estimates of between-level effects in multilevel data [92] and being unaffected by issues of collinearity or normality of predictors [94]. The generalized structural equation modeling (GSEM) function in Stata thus enabled us to best run multilevel structural equation models with the advantages discussed above [91]. Model selection using the GSEM function mainly considers the statistical significance of path coefficients and the theoretical relevance of the relationships [93]. We defined statistically significant effects as those with a p-value of less than 0.05, corresponding to a 95% confidence interval. Akaike Information Criterion (AIC) and Bayesian Information Criterion (BIC) can also be used for model selection and comparison since GSEM structural models do not produce absolute fit indices as indicated in previous research [93,94]. AIC serves to minimize the expected prediction error of the model, while BIC seeks to identify the true model if it exists within the comparison set [95]. Among multiple models, the one with the lowest AIC and BIC values indicates a better fit to the data [95].

Next, in line with the recommended steps for analyzing multilevel structural equation models, we estimated the null model and assessed whether there was significant clustering using the inter-class correlation coefficient (ICC) [92]. The ICC value was 0.02 (95% CI [0.00, 0.77]) which is less than 0.05 hence it does not indicate significant clustering [96]. We however continued with data analysis using the GSEM function despite the low ICC since some bias exists in any multi-level data [97].

### Ethics approval

Prior to data collection, we received institutional ethical approval from Strathmore University Institutional Scientific and Ethics Review Committee, (Reference number: SU-IERC1243/21) and national ethical approval from National Commission for Science, Technology & Innovation (Reference number: 747238). In line with the principles of research ethics, we assured anonymity of responses and written informed consent was obtained from all participants as provided in the cover letter that preceded the questionnaires.

## Results

### Descriptive analysis results

In terms of the SMEs characteristics, 48.50% of SMEs hailed from Nairobi County, 28.24% from Kiambu County and 23.26% from Nakuru County. The size of the SMEs was such that 46.52% had between 10 and 39 employees, 16.94% had between 40 and 69 employees and 36.54% had between 70 and 99 employees. In terms of sectoral representation, 79.07% were in the service industry while the rest were in the agricultural, mining and industrial sectors. The mode of working was predominantly remote (43.85%) while the mode of hybrid had 37.87% representation and physical mode had 18.27% representation. Lastly, 76.74% did not have information technology (IT) back-up facilities by the time the pandemic started in Kenya.

The mean scores for the variables under study were as follows. All variables under study achieved relatively strong mean scores with organizational resilience having a mean of 3.35, psychological capital had a mean of 3.00, transformational leadership had a mean of 3.05, and directive leadership had a mean of 3.18. Multivariate correlations between the variables show significant and positive correlations between organizational resilience, psychological capital and transformational & directive leadership styles. The means, standard deviations, and correlations are presented in Table 2.

**Table 2:** Descriptive statistics and correlations.

Constructs	Mean	S.D	1	2	3
**Organizational resilience**	3.35	0.88	–		
**Transformational leadership**	3.05	0.75	0.71^**^	–	
**Directive leadership**	3.18	0.93	0.66^**^	0.64^**^	–
**Psychological capital**	3.00	0.68	0.69^**^	0.74^**^	0.68^**^

** Correlation is significant at the 0.01 level (2-tailed).

SD, standard deviation.

### Structural model results

First, we examined the relationship between transformational leadership style and organizational resilience while controlling for the effects of the sector, presence of IT-back-up facilities and mode of working. We created dummy variables for the categorical variables taking particular interest in the service sector, physical working and presence of IT back-up facilities, all of which have been shown to have significant influence on organizational resilience during the pandemic period [73,76]. Results revealed a significant and positive relationship between transformational leadership (p < 0.0001, R^2^ =  0.82) and organizational resilience while controlling for the service sector, physical working and presence of IT back-up facilities. The results for the relationship between directive leadership and organizational resilience while controlling for the same factors was also significant and positive (p < 0.0001, R^2^ =  0.62) as demonstrated in the p value which is less than 0.05.

Additionally, the relationships between transformational (P < 0.0001 R^2^ =  0.67) and directive (P < 0.0001 R^2^ =  0.50) leadership styles and employees’ psychological capital were also significant and positive. The relationship between employees’ psychological capital and organizational resilience was also significant and positive (p < 0.0001, R^2^ =  0.88) while controlling for the service sector, physical working and presence of IT back-up facilities. Lastly, employees’ psychological capital mediated the positive relationship between transformational and directive leadership styles, and organizational resilience (p < 0.0001, R^2^ =  0.30) while controlling for the service sector, physical working and presence of IT back-up facilities. The confidence intervals of the mediation did not contain zero (95% CI [0.20, 0.41]) hence the mediation was significant [98]. The effect of leadership styles on organizational resilience did not completely disappear in the overall mediated model (for transformational was p < 0.0000, R^2^ =  0.42 & directive (p < 0.0000, R^2^ =  0.25) hence this was partial mediation [98]. The structural models including the overall model and model fit criteria are shown in Table 3.

**Table 3:** Structural model consolidated results.

Variables	DV: OGR	DV: OGR	DV: PsyCap	DV: PsyCap	DV: OGR	DV: OGR
	Model 1	Model 2	Model 3	Model 4	Model 5	Model 6
**Multilevel intercept**	0.74^***^ (0.12)	1.34^***^ (0.12)	0.94^***^ (0.08)	1.40^***^ (0.07)	0.62^***^ (0.14)	0.31^* ^(0.12)
**Transformational**	0.82^***^ (0.03)	–	0.67^***^ (0.02)	–	–	0.42^***^ (0.05)
**Directive**	–	0.62^***^ (0.03)	–	0.50^***^ (0.02)	–	0.25^***^ (0.03)
**PsyCap**	–	–	–	–	0.88^***^(.04)	0.30^***^(.05)
**Controls**	0.15^**^ (0.06)	0.13^*^ (0.07)	–	–	0.15^**^ (0.07)	0.11^**^ (0.06)
**Service sector**						
**Presence of IT back-up facility**	−0.02 (0.06)	−0.08 (0.07)	–	–	−0.01 (0.06)	−0.05 (0.05)
**Physical mode of working**	0.06 (0.07)	0.02 (0.07)	–	–	0.01 (0.07)	0.06 (0.06)
**Model fit criteria**	1131.03	1210.97	767.00	878.70	1169.21	1675.15
**AIC**						
**BIC**	1161.83	1241.78	784.60	896.30	1200.01	1732.35
**D.F**	7	7	4	4	7	13

N = 602.

Standard errors in parentheses.

Significance p < 0.1, *p < 0.05, **p < 0.01, ***p < 0.001.

OGR, organizational resilience; PsyCap, psychological capital; DF, degrees of freedom; DV, dependent variable.

In summary, as discussed above in a step-by-step manner, all hypotheses were supported as summarized in Table 4.

**Table 4:** Summary of decisions on the hypotheses.

Hypotheses	Verdict
H1: Transformational leadership is positively related to organizational resilience.	Supported
H2: Directive leadership is positively related to organizational resilience.	Supported
H3: Transformational leadership is positively related to employees’ psychological capital.	Supported
H4: Directive leadership is positively related to employees’ psychological capital.	Supported
H5: Employees’ psychological capital is positively related to organizational resilience.	Supported
H6: Employees’ psychological capital mediates the positive relationship between transformational leadership and organizational resilience.	Supported
H7: Employees’ psychological capital mediates the positive relationship between directive leadership and organizational resilience.	Supported

## Discussion

The present study investigated the link between leadership styles and organizational ability to cope with a crisis in a bid to find effective leadership during crises. Our research model was tested in the COVID-19 pandemic crisis context. We found that transformational and directive leadership styles had significant and positive relationships with organizational resilience. Consistent with literature, transformational leadership through emphasizing on individual and collective goals is crucial during change processes and crises [35,38]. Contrary to popular belief, directive leadership does not always imply the negativity of a highly authoritative leader who exercises absolute control over subordinates, but a directive orientation that involves competence, clarity and process monitoring that is crucial for SMEs during a crisis [15,41]. The present study therefore empirically supports the application of both transformational and directive leadership styles as effective leadership styles for achieving organizational resilience.

Additionally, we assessed the mediating effects of psychological capital on the relationship between leadership styles and organizational resilience. We found that the indirect relationship between transformational and directive leadership styles and organizational resilience was positively mediated by employees’ psychological capital. Consistent with previous literature, promoting organizational resilience should be the common goal orientation of both leaders and employees and this can be achieved by selecting an appropriate leadership style that improves the cognitive abilities of employees [99]. The present study thus indicates that psychological capital is an important resource during crises to alleviate job demands and facilitate organizations to be resilient. Consistent with past research, psychological capital acts as a positive characteristic in employees to overcome job strain and meet organizational objectives [34]. As organizational resilience has been shown to depend on multilevel interactions between leaders and employees, we proceed to discuss the theoretical and practical implications.

### Theoretical contribution

The present study contributes to the growing research on antecedents of organizational resilience [35,76,100]. Our theoretical framework combines three approaches of organizational resilience including the process, capabilities and multilevel approach. Firstly, we viewed organizational resilience as not merely about survival but also learning and bouncing back after crises which represents the process approach of organizational resilience [14,101]. Secondly, we based our study on two organizational resilience capabilities of planning and adapting which cover the behavioral and social aspects of organizational resilience [100]. Thirdly, we assessed individual level antecedents of organizational resilience in order to understand how resilience can be scaled up. Organizational resilience requires the interaction of individuals (employees and leaders) yet a holistic view of these multilevel antecedents was largely missing in literature [49,101].

There was also a gap in individual literatures (leadership and crisis management) to clearly understand which leadership styles are effective for promoting organizational resilience [76]. The present study bridged leadership and crisis management literature by analyzing how two positive leadership styles, namely, transformational leadership and directive leadership styles, relate to organizational resilience. Transformational leadership is popular in crisis management literature and directive leadership is highly used in practice during crises though it is not popular in both leadership and crisis management theory [102,103]. The present study found empirical support that both transformational and directive leadership styles have a positive relationship with organizational resilience. Thus the present study contributes to crisis-leadership literature which has tended to be mostly descriptive and conceptual based [14,104].

Regarding the mechanisms that explain how leaders build organizational resilience, previous literature had only conceptualized but not empirically tested employee commitment as a mediator on the relationship between shared leadership and organizational resilience [104], another research had conceptualized organizational values as an organizational level mediator between situational, transactional and transformational leadership and organizational resilience [14] and another had tested the mediating role of adaptive cultures on the relationship between transformational leadership and organizational resilience [35]. To the best of our knowledge, no study had focused on leader-follower psychological interaction, yet leaders do not operate alone to build organizational resilience in a crisis. Employees are also a critical source of organizational resilience as demonstrated in the mediation analysis in the present study.

In terms of the application of theories, the present study contributed by testing the JD-R theory which was applied in the present study to provide an appropriate conceptualization of the resilience of SMEs from a multilevel perspective [27]. The present study integrated personal resources into job resources as provided in the JD-R theory. This argument constituted the mediating mechanism of employees’ psychological capital on the relationship between leadership styles and organizational resilience. There were only a few studies that have integrated the extended JD-R model with both job and personal resources [27].

### Practical contribution

Crisis symbolizes two components, danger and opportunity [49]. Organizations can grow from their crisis experiences when they view adversity as an opportunity to recalibrate within a volatile environment [49]. The present study is based on the COVID-19 pandemic that provided an extensive crisis environment to develop and test organizational resilience models [105]. The present study contributes to practice by creating and testing a resilience framework for SMEs that capitalizes on frugal resources such as leadership styles and psychological capital [23]. A recurrent puzzling question is on how resilient organizations respond to crises differently than their less resilient counterparts [105]. By testing the link between leadership styles and employee’s psychological capital in a crisis, the present study offered a novel paradigm for SMEs in Kenya and similar contexts to understand how to develop their resilience capabilities.

The present study is also practically valuable for strategic human resources management because the results offer leaders with important empirical references for crisis- responsive HR management since more crises are likely in future [106]. The results of the present study can be used to reduce decision-making time in determining effective leadership styles in crises. Research had already identified specific competencies of effective leaders in healthcare organizations in crises such as enhancing communication [107] however specific leadership styles needed by SMEs were largely missing. The present study underscores the importance of leaders learning to be transformational by exemplifying a synergy of behaviors including individualized consideration, intellectual stimulation, inspirational motivation, idealized influence as well as to be directive by giving clear instructions to employees during crises. Leaders can certainly be made through training on appropriate behaviors [54].

The present study brought out the need for SMEs to invest in employees’ psychological capital. We found that organizational resilience is dependent on a pathway where leaders enhance employees’ psychological capital. Practically understanding this explanatory mechanism is crucial to inform HR policies that need to be adopted by SMEs to aid coordination between leaders and employees in order to build hope, efficacy, resilience and optimism. Positive mental dispositions have become increasingly important in investigating how employees can assist in positive adaptation to the environment [108]. Recognizing the malleable nature of psychological capital [52] can provide SMEs with better insight into the need to train leaders to develop their subordinates’ psychological capital. Notably, organizational resilience as pertains to SMEs largely is about the human involvement within organizations as one can hardly separate the SMEs from the people forming and operating them [109].

### Limitations and future research directions

In the present study, psychological capital only partially mediates the relationship between leadership styles and organizational resilience, indicating that there are likely other variables that also mediate this relationship. Future research should further explore the mechanisms underlying the effects of leadership styles on psychological capital from other theoretical perspectives.

The present study utilized non-probability sampling methods, in particular convenience sampling, because there is no comprehensive list of all SME employees in Kenya [64]. We acknowledge that convenience sampling method has limitations on the generalizability of the study findings [110] and recommend further probing of this research’s phenomena using probability sampling methods.

The present research also considered psychological capital as a higher order construct rather than based on its dimensions. Future studies could unpack the dimensions of psychological capital [51] to understand the nuances of how leadership styles influence hope, optimism, resilience and efficacy, to understand which aspects are most critical for organizational resilience.

We acknowledge the potential presence of common method bias because we employed the same data collection method for both exogenous and endogenous variables [75]. To evaluate and address some of the bias we followed recommendations by Podsakoff et al., [75] however future research can separate the data collection for the exogenous and endogenous variables in a longitudinal study. Also, given that the variables for the present study were collected at the same time using cross-sectional design, temporal precedence was not established and thus causality cannot be inferred from our results. Future research could collect longitudinal data to examine the causal relationship between variables as well as to establish temporal precedence of the mediating variable.

Finally, we know that organizational resilience requires the interaction of individuals (employees and leaders) sub-units (teams) and external partners (networks) yet a holistic view of these multilevel antecedents is largely missing [49]. Our study identified and tested two individual level predictors and an individual level mediator. There is therefore a need to assess team level (meso-level factors) antecedents of organizational resilience in future studies in response to the increasing importance of teams in organizations as well as to examine the role of external networks in achieving resilience.

## Conclusion

In these challenging times of crises, (e.g., pandemic, war and climate change) there is a growing momentum for not merely seeking profit but building resilience [49]. Despite the extant research on resilience and leadership, the necessity to infuse leadership with resilience and the call for its synthesis was triggered by the COVID-19 crises. Due to the amplified role of leaders in crisis and the need for timely action, we answered calls for more investigations on leadership during crisis [37]. In the present study, a novel attempt was made to advance the realm of organizational resilience among SMEs by studying a model involving transformational and directive leadership styles and psychological capital during a macro crisis. Since crises cannot be avoided, it is crucial for SME leaders to adopt effective leadership styles to better cope with future crises.

## Supporting information

S1 Fig Conceptual framework. (TIF)
