# Supplementary figures and images for: Surviving a crisis: A multilevel model of leadership styles, employees’ psychological capital and organizational resilience
Source: PLoS One. 2025 Feb 6;20(2):e0318515. doi: 10.1371/journal.pone.0318515 (PMC11801540; doi:10.1371/journal.pone.0318515)

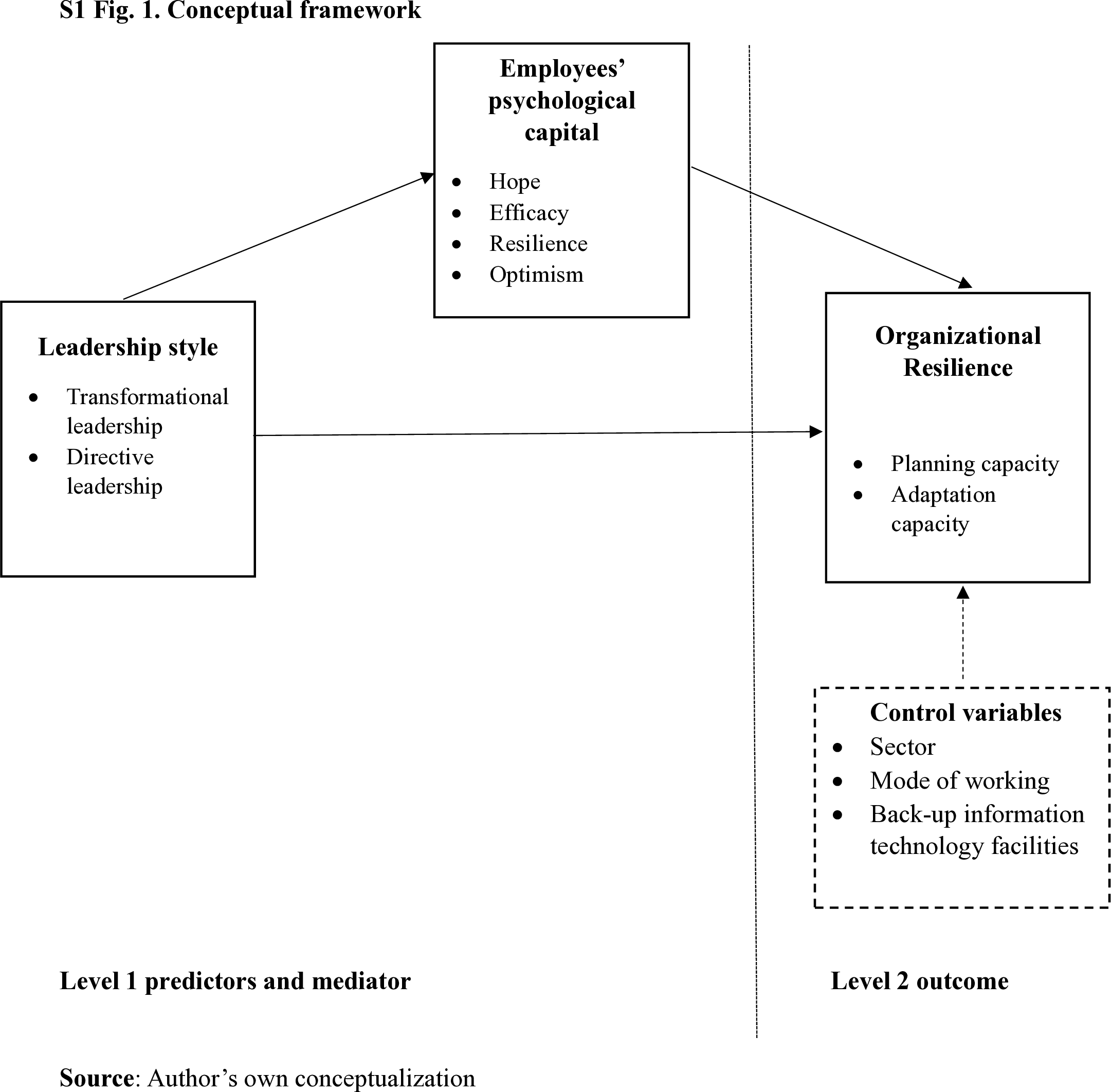

Supplement: S1 Fig — (TIF) [file pone.0318515.s001.tif]
